# An inducible CiliaGFP mouse model for *in vivo* visualization and analysis of cilia in live tissue

**DOI:** 10.1186/2046-2530-2-8

**Published:** 2013-07-03

**Authors:** Amber K O’Connor, Erik B Malarkey, Nicolas F Berbari, Mandy J Croyle, Courtney J Haycraft, P Darwin Bell, Peter Hohenstein, Robert A Kesterson, Bradley K Yoder

**Affiliations:** 1 1Department of Cell, Development, and Integrative Biology, University of Alabama at Birmingham Medical School, Birmingham, AL 35294, USA; 2 Department of Craniofacial Biology, Medical University of South Carolina, Charleston, SC 29425, USA; 3 Ralph H. Johnson Veterans Administration Medical Center, Department of Medicine, Division of Nephrology, Medical University of South Carolina, Charleston, SC 29425, USA; 4 The Roslin Institute, University of Edinburgh, Easter Bush Campus, Midlothian Scotland, UK; 5 5Department of Genetics, University of Alabama at Birmingham Medical School, Birmingham, AL 35294, USA; 6 6Center for Translational Science, Children’s National Medical Center, Washington, DC 20010, USA

**Keywords:** Somatostatin receptor 3, ROSA26 locus, Inducible transgene, In vivo cilia labeling, Intravital microscopy

## Abstract

**Background:**

Cilia are found on nearly every cell type in the mammalian body, and have been historically classified as either motile or immotile. Motile cilia are important for fluid and cellular movement; however, the roles of non-motile or primary cilia in most tissues remain unknown. Several genetic syndromes, called the ciliopathies, are associated with defects in cilia structure or function and have a wide range of clinical presentations. Much of what we know about the formation and maintenance of cilia comes from model systems like *C. elegans* and *Chalmydomonas*. Studies of mammalian cilia in live tissues have been hampered by difficulty visualizing them.

**Results:**

To facilitate analyses of mammalian cilia function we generated an inducible Cilia^GFP^ mouse by targeting mouse cDNA encoding a cilia-localized protein somatostatin receptor 3 fused to GFP (Sstr3::GFP) into the ROSA26 locus. In this system, Sstr3::GFP is expressed from the ubiquitous ROSA26 promoter after Cre mediated deletion of an upstream Neo cassette flanked by lox P sites. Fluorescent cilia labeling was observed in a variety of live tissues and after fixation. Both cell-type specific and temporally regulated cilia labeling were obtained using multiple Cre lines. The analysis of renal cilia in anesthetized live mice demonstrates that cilia commonly lay nearly parallel to the apical surface of the tubule. In contrast, in more deeply anesthetized mice the cilia display a synchronized, repetitive oscillation that ceases upon death, suggesting a relationship to heart beat, blood pressure or glomerular filtration.

**Conclusions:**

The ability to visualize cilia in live samples within the Cilia^GFP^ mouse will greatly aid studies of ciliary function. This mouse will be useful for *in vivo* genetic and pharmacological screens to assess pathways regulating cilia motility, signaling, assembly, trafficking, resorption and length control and to study cilia regulated physiology in relation to ciliopathy phenotypes.

## Background

Cilia have essential roles in regulating signaling pathways that control development and normal tissue function (for recent reviews see [1-3]). There are two basic types of cilia. The first is the primary, non-motile cilium that is composed of a ring of nine microtubule doublets ensheathed by a ciliary membrane and found one per cell. The second type is the motile cilium, which has an additional central pair of microtubules and has a synchronized beat that propels fluid. Motile cilia include those in the trachea and ependymal cells of the ventricles in brain.

Cilia are unique sub-domains of the cell comprised of specific proteins and signaling molecules [4]. Several key signaling pathways are now known to be regulated by the cilium, and numerous G-protein coupled receptors, channels and transcription factors specifically localize to the cilium [2]. As such, a group of syndromes, called the ciliopathies, are caused by defects in the structure or function of the cilium. Ciliopathy disease states range from mild, where only one or a few organ systems are affected, such as in Leber’s Congenital Amaurosis, to more severe syndromes involving nearly all organ systems and resulting in perinatal lethality, as seen in Meckel-Grüber Syndrome. Several phenotypes are related specifically to motile cilia defects, such as chronic bronchiectasis, altered left-right body axis specification, infertility and hydrocephalus. Other ciliopathy phenotypes are linked to defects in ciliary signaling or sensory roles leading to blindness, anosmia, polydactyly, obesity or polycystic kidney disease [1-3].

Model organisms, such as *Caenorhabditis elegans* and *Chlamydomonas rheinhardtii,* have been used extensively to study ciliary biology. These systems have the advantages of rapid genetic screens and biochemical analysis of cilia proteins, but perhaps most importantly the cilia in these models are readily visible in living samples. As such, these organisms have contributed greatly to our general understanding of cilia formation and maintenance. However, understanding the physiological roles for mammalian cilia in most tissues and the molecular mechanisms behind ciliopathy associated diseases remains a challenge in the field. Unfortunately, to date there have been no inducible, *in vivo* mammalian models to easily visualize cilia.

To address this limitation, we generated the Cilia^GFP^ mouse that allows direct visualization of mammalian cilia *in vivo*. Cilia labeling is inducible using Cre recombinase for tissue or cell type specific analysis or the label can be constitutively expressed. We show here the utility of the Cilia^GFP^ mouse for *in vivo* and *ex vivo* detection and analysis of cilia. Importantly, we document here a novel finding showing synchronized movement of primary cilia in the kidney of a live mouse that underscores the utility of this model.

## Methods

### Construct generation and mouse engineering

**Figure 1:**
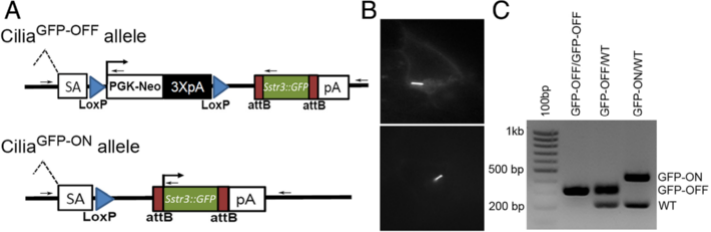
**Sstr3::GFP ROSA26 targeted alleles.** (**A**) Schematic of the Sstr3::GFP conditional (Cilia^GFP-OFF^) and expressing (Cilia^GFP-ON^) alleles. Before Cre mediated deletion (Cilia^GFP-OFF^), the expression of Sstr3::GFP is precluded by the PGK promoted Neo cassette flanked by LoxP sites (blue triangles). After Cre mediated recombination (Cilia^GFP-ON^) at the LoxP sites the Neo cassette is deleted allowing the expression of Sstr3::GFP. Splice acceptor (SA), blue triangles represent LoxP sites, PKG-Neo: Phosphoglycerate kinase promoter driving a neomycin resistance gene followed by three polyA tail signals (3XpA, black). The remaining attB sites after Gateway recombination are represented by maroon boxes flanking the cDNA of Sstr3::GFP. (**B**) To confirm expression and ciliary localization, embryonic stem (ES) cells with correctly targeted Sstr3::GFP were differentiated and transduced with a Cre expressing lentivirus to induce expression of Sstr3::GFP. (**C**) A four primer combination was used for genotyping by PCR to amplify the wild type (226 bp), Cilia^GFP-OFF^ (317 bp) and Cilia^GFP-ON^ (423 bp) alleles. Lane 1:100 bp ladder, lane 2: Cilia^GFP-OFF^/Cilia^GFP-OFF^, lane 3: Cilia^GFP-OFF^/WT, and lane 4: Cilia^GFP-ON^/WT. Primer binding sites represented in (**A**) as small black arrows, sequences in Methods.

To generate the Sstr3::GFP ROSA26 targeting construct, Sstr3::GFP was amplified from an expression vector (a kind gift from Dr. Pazour, University of Massachusetts) with primers adding attB sites to the 3′ and 5′ ends, which was then cloned into the ROSA26 targeting vector (pRosa26DEST, Addgene #21189) using Gateway cloning technology (Life Technologies, Grand Island, NY, USA) (Figure 1A) [5]. Primers, forward: ggggacaagtttgtacaaaaaagcaggcttaaccatggccactgttacctatccc, and reverse: ggggaccactttgtacaagaaagctgggtattacttgtacagctcgtccatgcc. The Sstr3::GFP construct was electroporated into Primogenix B6 (C57BL/6 N-tac) embryonic stem (ES) cells and G418 resistant colonies were established as described previously ([6,7]). The ES cell colonies were screened by long range PCR using forward primer: aaaagcagcagccatctgagatag, and reverse: cgagggacctaataacttcgtatagc, to yield a 2.4 kb product. To test expression and ciliary localization prior to generating the mice, the targeted ES cells were differentiated by removing leukemia inhibiting factor and serum from the media. Cells were subsequently transduced with a Cre lenti virus to remove the Neo cassette and activate the expression of Sstr3::GFP (Figure 1B). Correctly targeted ES cells were injected into blastocysts to generate male chimeras. Germline passage was obtained by crossing chimeric males to albino C57BL/6 females. Genotyping primer areas follow: a common forward within the 5′ homology arm: ctcgtgatctgcaactccag; a reverse in the 3′ homology arm: gctgcataaaccccagatgactcc; a reverse near the 5′ PKG-Neo site: gcgcatgctccagactgccttg; and a reverse at the 5′ end of Sstr3: gcggatgtgttccccagggtgg (Figure 1A, arrows). Together the primers yield a 226 bp wild type band, a 317 bp product from the targeted allele containing the floxed-stop-sequence (Cilia^GFP-OFF^), and a 423 bp band that is amplified after excision of the floxed-stop cassette (Cilia^GFP-ON^) (Figure 1C). Systemic and cell type specific deletion of the Neo gene was accomplished using Adenovirus EIIa early promoter Cre (Tg(EIIa-cre)C5379Lmgd, [8] hereafter called EIIa Cre); Proopiomelanocortin Cre (POMC, Tg(Pomc1-cre)1Gsb, [9] hereafter called POMC Cre), and the Pancreatic and Duodenal Homeobox Cre ER (Tg(Pdx1-cre/Esr1*) 1 Mga [10], hereafter called Pdx1Cre ER). Germline, systemic expression of the Cilia^GFP-ON^ allele was accomplished crossing female EIIa Cre; Sstr3::GFP with wild type C57BL/6 mice. This study was carried out in accordance with the recommendations in the Guide for the Care and Use of Laboratory Animals by the National Institutes of Health. The protocols used were approved and conducted according to the University of Alabama at Birmingham IACUC.

### Tamoxifen injections

To activate the Cre recombinase in Pdx1CreER animals, mice were injected with 6 mg of tamoxifen every other day over a five-day period and the pancreata were harvested three days after the final injection. Tamoxifen (Sigma-Aldrich, St. Louis, MO T5648) was dissolved in corn oil at 20 mg/ml, and bolus injections of 300 μl were administered intraperitoneally.

### Renal tubule isolation

Mice were anesthetized and euthanized, and whole kidneys were isolated and cut into approximately 2 mm pieces. The tissue was incubated at 37°C in three changes of collagenase buffer for 30-minute intervals with gentle agitation every 10 minutes (Collagenase buffer: 10 mg/ml Collagenase Type IV (Sigma #C5138), 50 U/mL DNAse I (Sigma #D5025), and 0.1 mg/ml Soybean Trypsin Inhibitor (Life Technologies, Grand Island, NY #17075-029) in DMEM/F12). After each 30-minute incubation, the supernatant was removed and replaced with fresh collagenase buffer. The supernatants, containing the tubules, were gently pelleted at 100 Relative Centrifugal Force (RCF) for one minute, the tubules were washed three times in phosphate buffered saline (PBS) containing 3% bovine serum albumin (BSA) and pooled. The tubules were kept on ice and imaged live or were fixed in 4% paraformaldehyde (PFA) for five minutes followed by blocking and processing for immunofluorescence analysis.

### *Ex vivo* live imaging

Brains from Cilia^GFP^ EIIa Cre mice were removed after anesthetization and decapitation. The *ex vivo* brains were cut sagittally down the midline and placed cut surface down on cover glass in room temperature sterile filtered, artificial cerebro-spinal fluid (125 mM NaCl, 2.5 mM KCl, 1.25 mM NaH_2_PO_4_, 2 mM CaCl_2_, 1 mM MgCl_2_, 25 mM NaHCO_3_, 25 mM glucose, pH 7.3). Cilia were imaged live using a Hamamatsu C9100-50 EM-CCD camera (Hamamatsu Photonics K.K., Hamamatsu City, Japan) on an inverted Nikon TE2000-U microscope equipped with a 60× Plan Apochromat oil-immersion TIRFM objective (numerical aperture (NA), 1.49; Nikon Instruments Inc., Melville, NY), and a Perkin Elmer Ultraview-ERS 6FE spinning disk confocal module controlled by Volocity 6.2 software (Perkin Elmer, Shelton, CT, USA). Visualization of Sstr3::GFP was accomplished using a fluorescein isothiocyanate (FITC) laser filter set (Chroma Technology, Rockingham, VT, USA); the light source was a 200 mW 488 nm argon laser (Melles Griot, Carlsbad, CA, USA). For immunocytochemistry, we imaged Alexa-594 conjugated antibody staining using a rhodamine/TRITC filter set (Chroma) and 20 mW 568 nm argon krypton laser (Melles Griot), Alexa-647 conjugated antibody staining using a Cy5 filter set (Qioptiq Inc., Fairport, NY) and 15 mW 640 nm diode laser (Melles Griot), Hoechst staining using a 4',6-diamidino-2-phenylindole (DAPI) filter set (Chroma) and 15 mW 405 nm diode laser (Qioptiq).

### *In vivo* analysis of renal cilia

A group of 8- to 16-week-old mice were anesthetized by intraperitoneal injection of 2.5% tribromoethanol (Sigma T48402). A dorsal incision was made just rostral to the iliac crest and the kidney was gently teased out through the opening, making sure not to damage the renal artery, vein or ureter. The mouse was placed, incision side down, inside a heated chamber maintained at 37°C (LiveCell™ Stage Top Incubation System #05-11-0035, Pathology Devices, Inc., Westminster, MD, USA) with the kidney positioned on a coverslip bathed in PBS. The mice were continuously administered isoflurane at 1 to 2% (VetOne Boise, ID#13985-030-60) throughout the procedure and were euthanized after imaging.

### Tissue isolation and sectioning

Fresh tissues were isolated and processed as described in [11]. For immunofluorescence detection of neuronal cilia, animals were anesthetized with 2.5% tribromoethanol (Sigma T48402), and transcardially perfused with PBS, followed by 4% PFA. The brain was then isolated and processed in the same manner as other tissues. Tissues were dissected into PBS, fixed in 4% PFA overnight at 4°C, saturated with 30% sucrose in PBS, oriented in optimal cutting temperature compound (Fisher Scientific, Pittsburgh, PA #14-373-65), and frozen in a dry ice-ethanol bath. Sections 10 to 15 μM thick were cut and mounted onto slides, fixed again in 4% PFA for five minutes at room temperature (RT) and then used for immunofluorescence staining. Sperm were collected by removing epididymides and incubating in Whittens-HEPES buffer (100 NaCl, 4.4 KCl, 1.2 KH2PO4, 1.2 Mg SO4, 5.4 glucose, 0.8 pyruvate, 4.8 lactate, 20 HEPES in mM) for 10 minutes and then capacitated by incubation in Whittens-HEPES supplemented with 15 mM NaHCO3 and 5 mg/ml BSA for 1 hour.

### Immunofluorescence analysis

For immunofluorescence detection of cilia, staining was performed as in [12]. Briefly, cryosections were blocked in PBS+ (1% BSA, 1% Normal Donkey Serum, in PBS + 0.1% Triton-X 100) for 30 minutes. Primary and secondary antibodies were diluted in blocking solution and applied to sections for one hour at RT or overnight at 4°C. After incubation, the slides were washed in PBS three times for five minutes each before being stained with Hoechst, and mounted with 1,4-diazabicyclo[2.2.2]octane (DABCO). The following primary antibodies were used: acetylated α-tubulin (Sigma T7451, 1 mg/ml input) pre-conjugated to AlexaFluor 647 (Life Technologies #A-20186) and used at 1:2,000, Adenylyl cyclase III (ACIII, Santa Cruz Biotechnology, Santa Cruz, CA, USA, #sc-11617) used at 1:500, and Arl13b (a gift from Dr. Tamara Caspary at Emory University) used at 1:2,000. The following secondary antibodies were used: anti-goat and anti-rabbit AlexaFluor-594 each used at 1:1,000.

For isolated tubules, immediately upon isolation the tubules were fixed in 4% PFA for 5 minutes followed by blocking in PBS+ for 30 minutes. Tubules were incubated with the direct conjugated acetylated α-tubulin antibody for 30 minutes at RT and washed in PBS for 5 minutes. Nuclei were stained with Hoechst (Sigma #33258), and imaged in PBS or 50% glycerol droplets on cover glass.

## Results and discussion

Cilia^GFP^ mice were engineered to express the cilia localized somatostatin receptor 3 protein fused with green fluorescent protein (Sstr3::GFP). Since the construct was targeted into the ubiquitously expressed ROSA26 locus it should permit labeling of cilia in nearly every tissue or cell type in the body depending on the Cre line used to delete the floxed Neomycin cassette (Figure 1). The utility of the Cilia^GFP^ model was assessed using ubiquitous and cell type-specific Cre lines in fixed tissue, in live *ex vivo* samples, and *in situ* in the context of living animals. In Cre negative control animals (Cilia^GFP-OFF^/WT;Cre-) no GFP labeling was evident (Additional file 1: Figure S1A-C). However, it should be noted that the level of activation of the ROSA26 locus has been reported to decrease in postnatal animals, and to show minimal activation in a few cell types, such as astrocytes [13], which may impact the utility of this mouse model in certain circumstances.

### The brain

The ependymal cells lining the ventricles are important for movement of cerebral spinal fluid and defects in their motility are associated with hydrocephalus [14,15]. Interestingly, the clinical importance of primary cilia in the brain has recently become apparent, despite a general lack of understanding of their function [1,16]. On neurons, disruption of the primary cilium or ciliary proteins revealed that they have an important role in regulating satiation and object recognition through unknown mechanisms [16-18]. In addition, cilia are known to be important for sight and smell [16,19-21] and recently it was demonstrated that they regulate pathways involved in adult neurogenesis and migration of newborn neurons [22]. However, the ubiquitous nature of primary cilia on most neurons in the central nervous system (CNS) was unexpected [23]. While the most common method of cilia visualization in mammals relies on antibodies against post-translationally modified forms of tubulin, such as acetylated α-tubulin, or polyglutamylated tubulin, these methods prove especially challenging in the brain since tubulin throughout neuronal processes and cell bodies are highly post-translationally modified [24]. This has made visualizing cilia on neurons difficult without special fixation and perfusion conditions, thus this new cilia label mouse model will simplify and streamline studies of neuronal cilia.

**Figure 2:**
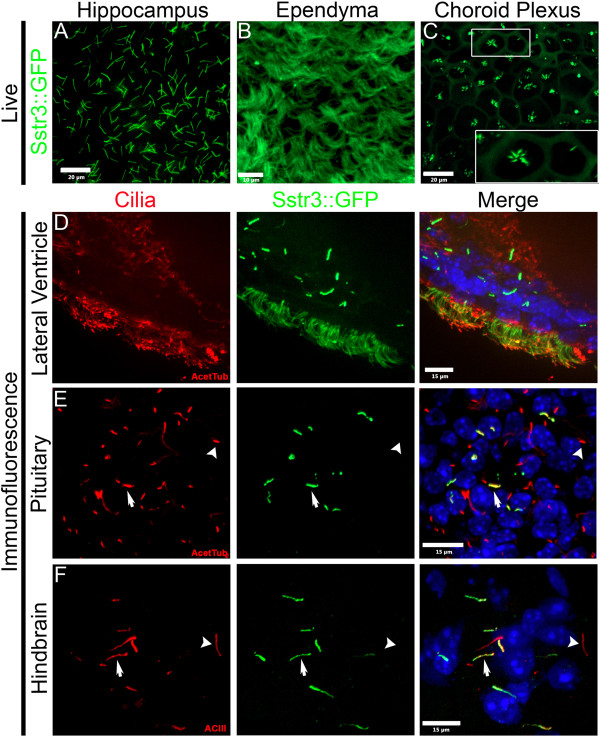
**Analysis of Sstr3::GFP expression in the brain.** (**A-C**) Images from *ex vivo* whole mount brains. Sstr3::GFP signal (green) in (**A**) hippocampal primary cilia, (**B**) ependymal motile cilia and (**C**) choroid plexus cilia. (**D-F**) Sstr3:GFP co-localization with known ciliary markers. (**D**) Motile cilia of the lateral ventricle, (**E**) pituitary primary cilia, and (**F**) hindbrain primary cilia. Cilia were immunolabeled (red) with either acetylated α-tubulin (AcetTub) in **D** and **E** or adenylate cyclase III (ACIII). Nuclei were stained with Hoechst (blue). Arrows indicate cilia that are double-labeled and arrowheads indicate cilia that are GFP negative in agreement with mosaic EIIa Cre activity.

To determine whether the Cilia^GFP^ mouse could be utilized for visualizing neuronal cilia in live *ex vivo* samples, we analyzed EIIa Cre; Cilia^GFP^ mice with mosaic activation of the Sstr3::GFP (Cilia^GFP-ON^) allele [8]. Primary cilia were easily detected on cells throughout the brain. For example, primary cilia were found in the hippocampus (Figure 2A) and motile cilia were detected on the ependymal cells within the ventricles (Figure 2B). On the choroid plexus epithelial cells, cilia are usually found in small groups or a single cilium per cell (Figure 2C inset) [14].

Time-lapse live imaging was performed on the ependymal cells to explore the potential utility of the Cilia^GFP^ mouse in cilia motility studies. Images were captured at 15 frames per second, and clear and distinct cilia could be seen (Additional file 2, see Methods). All of these cilia were visualized in live samples immediately after isolation with little or no preparation and no counterstaining (Figure 2A-C, and Additional file 2).

In addition to live imaging, the GFP label was easily visible after fixation and subsequent immunofluorescence staining with markers used to confirm that Sstr3::GFP labels cilia. In the lining of the lateral ventricle of Cilia^GFP-ON^ Cilia^GFP^ mice, Sstr3::GFP and acetylated α-tubulin co-localize within many of the ependymal cells and in the primary cilia of neighboring cells (Figure 2D). Interestingly, the primary cilia labeling is somewhat brighter than it is on the motile ependymal cilia; this is likely due to the increased number of cilia resulting in greater membrane surface area within which the Sstr3::GFP label can become diluted. This has been observed for the hedgehog pathway mediator, Smoothened, in cells induced to form multiple primary cilia [25]. Similar primary cilia staining was observed in the pituitary (Figure 2E); and in the hindbrain region (Figure 2F) where the GFP signal co-localized with acetylated α-tubulin and the neuronal cilia marker ACIII, respectively. Together these images, as well as those in the living samples, demonstrate that the expression levels in the brain are sufficient to facilitate studies of neuronal cilia. For example, this model would facilitate the analysis of the prevalence of cilia, differences in cilia morphology, or assessment of the efficiency of cilia ablation in specific brain regions (for example, after Cre deletion of a floxed ciliary allele). Also, this model would enhance studies requiring live tissues, such as the measurement of ligand induced translocation of proteins into and out of the cilium, in electrophysiology to patch ciliated neurons or even the cilium itself, or in pharmacological studies of factors regulating cilia length dynamics in the brain.

### The kidney

**Figure 3:**
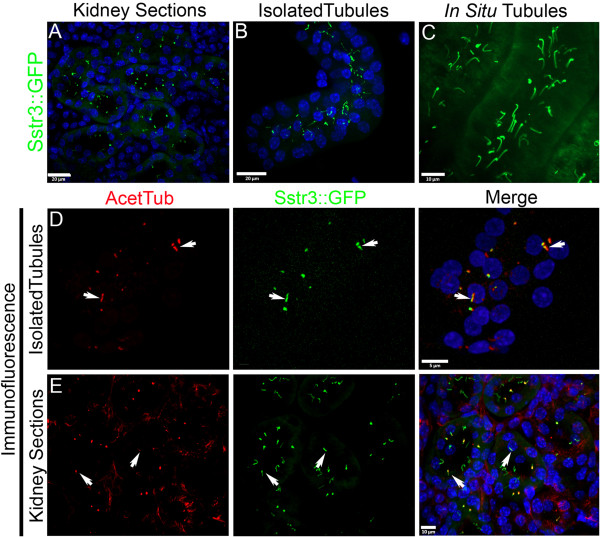
**Sstr3::GFP labeled cilia in the kidney.** Sstr3::GFP signal (green) in (**A**) fixed cryosections of the kidney, (**B**) isolated EIIa Cre; Cilia^GFP^tubules, (**C**) *in situ* in the proximal tubule of an anesthetized mouse. Immunofluorescence staining of (**D**) isolated tubules and (**E**) kidney sections and Sstr3:GFP localization. Acetylated α-tubulin (red) labeling was used to validate cilia localization of the Sstr3::GFP fusion protein. Nuclei are labeled with Hoescht (blue). Arrows indicate examples of cilia that are double-labeled.

The formation of cysts in the kidney is a common pathological feature associated with multiple human ciliopathies, including forms of polycystic kidney disease caused by mutations in polycystin 1 and polycystin 2, where cilia are present [1,16]. Although the causes are not yet known, significant effort has gone into determining how the disruption of cilia function results in cystogenesis. Under normal conditions, the renal cilium is thought to be a mechanosensor, wherein deflections of the ciliary axoneme by fluid flow elicits a cytosolic calcium response [26]. *In vitro* cell culture studies have determined that this mechanosensory response is impaired in cilia mutants, as well as in mutants lacking polycystin-1 or polycystin-2 [27]. These data lead to a model that cysts develop through loss of the mechanosensory signal; however, to date, *in vivo* studies validating this hypothesis have not been performed. To evaluate whether the Cilia^GFP^ mouse will be useful in visualizing cilia in the kidney and to address clinically important questions, such as whether or not flow induces ciliary deflection, we analyzed the expression of Sstr3::GFP in EIIa Cre kidneys (Figure 3). In fixed samples, acutely isolated tissue and live animals, cilia could easily be detected making studies of cilia length, orientation, motility and analysis of the whole tubule/kidney practical. However, it should be noted that we are overexpressing a cilium-localized receptor that has been shown to alter the length and morphology of the primary cilium in some cases [28].

In kidney sections, in live isolated tubules, and *in vivo****,*** GFP-labeled cilia were readily identifiable (Figure 3A-C). Again the label was seen without fixation or staining and persisted throughout handling and imaging. In isolated tubules, many cilia remained attached through isolation, fixation, staining and imaging (Figure 3D). However, some GFP labeled debris was observed in the isolated tubules that are likely to be ciliary fragments broken off during isolation. These fragments were not observed in the imaging of intact kidneys. Again, the specificity of ciliary localization was confirmed in tubules and in sections using acetylated α-tubulin (Figure 3D, E).

**Figure 4:**
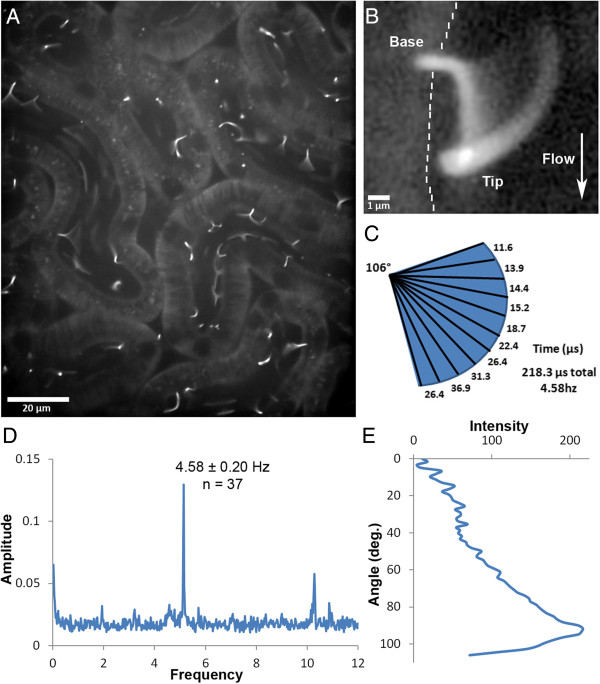
**Analysis of ciliary movement in the kidney.** (**A**) Sstr3::GFP-expressing cilia in kidney proximal tubules in a live mouse. Image is the average fluorescence over 40 s displaying the full range of motion of the cilia. (**B**) Three dimensional confocal rendering of the sweep of a single cilium over time. The wall of the tubule is indicated by a dashed line and direction of fluid flow by the arrow. (**C**) Graphic representation of the cilium sweep in (**B**) measuring the total angle traveled and the proportion of time the cilium spent in each 10^th^ of the total path during an oscillation cycle. Calculations based on (**D**) and (**E**). (**D**) Fast Fourier Transform analysis of the motion of cilia showing a uniform 4.58 Hz oscillation. (**E**) Line-scan of the fluorescence intensity along the path swept out by the tip of the cilium in (**B**).

Next, using *in situ* imaging techniques we evaluated cilia in the kidneys of live EIIa Cre: Cilia^GFP^ mice. Primary cilia could be clearly observed within the proximal tubules of the cortex (Figure 3C). In the live mice, the cilia did not simply protrude into the lumen perpendicular to the wall of the tubule; instead they all bend in the same direction nearly parallel to the apical surface along the length of the tubule. These cilia remain bent in this position with an occasional cilium reversing its position (Additional file 3). The deflection of the cilium is likely due to the large amount of primary filtrate passing through the proximal tubules. Interestingly, most of the cilia appear to be bending at a regular and specific point above the base and when oscillating cilia are observed, they generally move in an arc of approximately 106° (Figure 4C). Similar observations have been made using *in vitro* flow studies and have been attributed to the rigidity of the microtubules within the ciliary axoneme [29]. Modeling the cilium as an elastic cantilevered column fixed at the base results in the same bending profile when subjected to flow induced shear stress [30-33]. Alternatively, this bend may be attributable to a molecular domain such as the transition zone, the inversin compartment [34,35], or being embedded within the ciliary pocket [36].

Another interesting observation made in 5 out of 11 mice analyzed was that the cilia within a nephron would oscillate back and forth within the tubular lumen (Figure 4A, B and Additional file 4). This oscillation was rapid (4.58 ± 0.2 Hz, Figure 4D) and could be captured using relatively high speed image acquisition (approximately 26 fps). The sweep of the cilium during oscillation was irregular, where during each oscillation the cilium would spend the majority of the time bent along the tubule wall compared to any other point of its sweep (Figure 4C, E). These oscillations are most likely passive and not a result of molecular motors, such as dynein, as there is no evidence that these primary cilia have the machinery necessary for active motility [37]. In addition, the frequency of oscillation is similar to that documented for mouse heart rates under anesthesia [38]. Also, the presence of this oscillatory motion would change in the same animal over time, either appearing or disappearing during the course of the experiment, which suggests that the movement may be elicited by the depth of anesthesia, heart rate, stroke volume, blood pressure and their impact on glomerular filtration; however, we were unable to simultaneously measure the heart rate of mice while imaging. Furthermore, most of the cilia in a whole field move in unison, suggesting regulation at the level of the whole kidney, not at the level of the individual cells/tubules/glomeruli. Additional evidence supporting a passive mechanism is that the movement of the cilium in the tubules stops almost immediately upon death and the cilia extend nearly perpendicularly into the nephron lumen (Additional file 5, N = 2). Together these data suggest that tubular flow is not constant. Regular periodic oscillation in the flow rate of glomerular filtrate has been documented using fluorescent dextran [39] along with observations of oscillation in proximal tubular pressure [40]. This pulsatile flow rate in the proximal tubules provides a mechanism for the oscillation of cilia that we observe and also explains why the cilia spend a large proportion of each oscillatory sweep bent in the downstream position.

An alternate explanation is that the cilia, at least in the proximal tubule images captured here, are not passive but actually exhibit motile behavior. It should be noted that some studies have found that cilia in the node of the gastrulation stage mouse embryo have a 9 + 0 structure (similar to the kidney) and have a rotational motility that is distinct from the waveform motion of cilia on ependymal or tracheal cells [41,42]. However, others studies have reported that the node has a second form of cilia that has a 9 + 2 arrangement and it is not clear which form is actually responsible for the rotational beating [43]. Follow-up studies will be necessary to conclusively determine the cause of the ciliary oscillation which could impact our understanding of ciliopathy disorders, such as polycystic kidney disease.

### The eye

**Figure 5:**
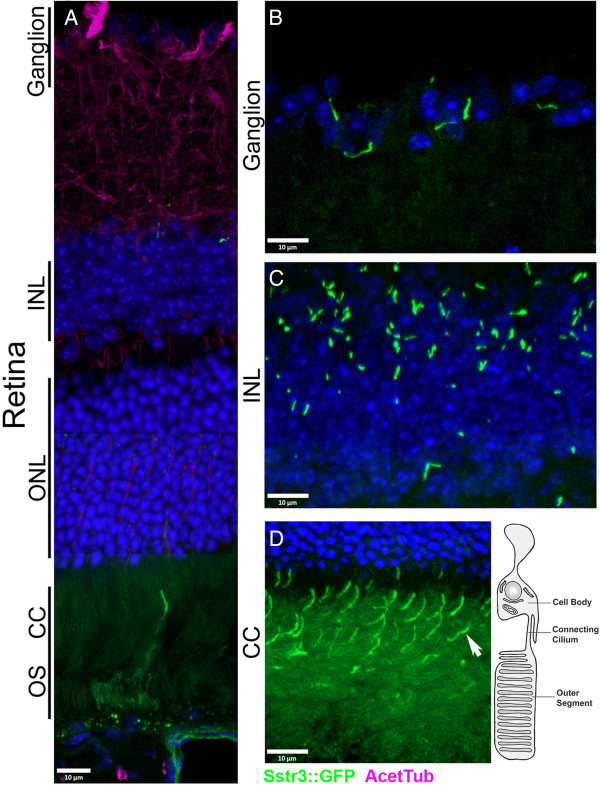
**Sstr3::GFP localization within the retina.** (**A-D**) Sstr3::GFP localization in the mouse retina. Sstr3::GFP signal (green) seen (**A**) throughout the retina, (**B**) in cilia of ganglion cells in the ganglion layer, (**C**) in cilia of cells (bipolar, horizontal, amacrine or müller cells) of the inner nuclear layer (INL), and (**D**) in the connecting cilium (CC) of rod cells. For orientation, a rod cell is depicted in the schematic on the right. The section in A was immunolabeled with antibodies to acetylated α-tubulin (purple), nuclei are labeled with Hoescht (blue). CC, Connecting cilium; NL, inner nuclear layer; ONL, outer nuclear layer; OS, outer segment. Arrow indicates an example of Sstr3::GFP containing connecting cilium, anterior is up.

Loss of vision is also associated with multiple ciliopathies, such as Senior-Løken syndrome, Leber’s Congenital Amaurosis and Bardet–Biedl syndrome [19,44,45]. This is due to dysfunction in the structure or trafficking at the connecting cilium (CC), a highly modified primary cilium in the rod and cone photoreceptors of the retina (Figure 5D, diagram) [19]. Defects in trafficking, protein turnover, ciliary assembly or the distribution of the signaling components required for vision are all associated with retinal degeneration [1,19]. Due to the stereotypic anatomy of the retina, and the exaggerated ciliary structure (Figure 5D), the rod cells in the retina are a useful model of ciliary function and trafficking [19,46,47], thus endogenous ciliary labeling would be beneficial for longitudinal *in vivo* studies.

To determine if the Cilia^GFP^mouse would be sufficient to analyze the photoreceptor CC, we evaluated the retinas of EIIa Cre; Cilia^GFP^ mice. Interestingly, the ganglion cells of the retina contained many ciliated cells (Figure 5B) as did many of the cells in the anterior region of the inner nuclear layer (INL, Figure 5C). GFP was concentrated in the CC of the photoreceptors but is detectable in the outer segments (Figure 5D, Additional file 1: Figure S1F). In addition, rhodopsin staining indicated that the Cilia^GFP^ label does not overtly interrupt trafficking of rhodopsin or affect the health of the outer segment (Additional file 1: Figure S1F). Finally, attempting to label the cilia in the retina with ciliary markers, such as Arl13b and acetylated α-tubulin, frequently requires antigen retrieval and can be challenging using standard immunofluorescence protocols (Figure 5, acetylated α-tubulin in purple). Thus, the Cilia^GFP^ mouse will be useful for identifying the connecting cilia in the retina of live mice, and in samples without relying on antibody staining approaches.

### Spatial and temporal control of expression

**Figure 6:**
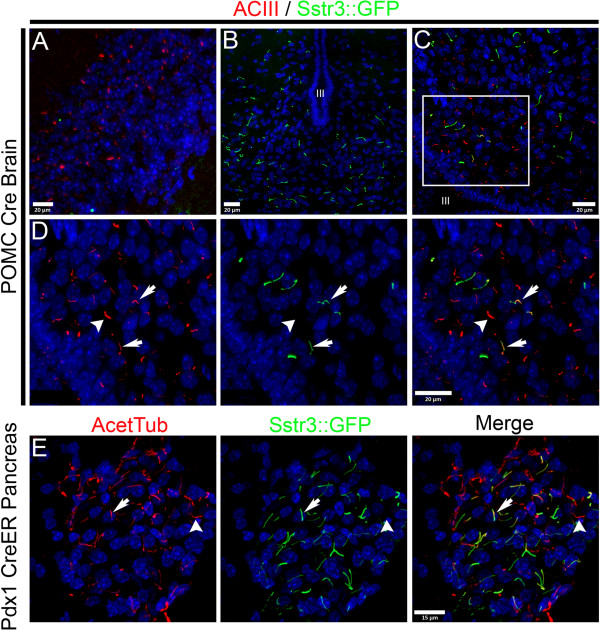
**Cell type specific labeling of cilia using Cre mediated activation in the Cilia**^**GFP **^**mouse.** (**A**) Control section of the dentate gyrus in a POMC Cre; Cilia^GFP^ mouse showing lack of green Sstr3::GFP containing cilia. Cilia are identified by immunofluorescence analysis with ACIII (red). (**B-D**) Sstr3::GFP cilia (green) are seen in the area of the arcuate nuclei (ARC) of the hypothalamus surrounding the third ventricle (III) in POMC Cre; Cilia^GFP^ mice. Cilia in the ARC were identified by immunofluorescence analysis with ACIII (red) in (**C** and **D**). Examples of dual labeled ACIII (red) and Sstr3::GFP (green) positive cilia are indicated by arrows, an example of a cilium labeled with ACIII but not Sstr3::GFP that is likely on non-POMC neurons is indicated by an arrowhead. (**E**) Sstr3::GFP expression in cilia on pancreatic islet cells from a Pdx1 CreER; Cilia^GFP^ mouse after tamoxifen induction. Cilia are labeled with (E) acetylated α-tubulin (Acet Tub) in red. Many cilia were positive for both Sstr3::GFP and acetylated α-tubulin (arrows) while other cilia did not possess Sstr3::GFP and are likely non-β-cells (arrowhead).

Having the ability to label cilia on a specific cell type *in vivo* will facilitate studies to address what roles cilia have on these cells in different tissues. Our previous work has shown that primary cilia on a subset of neurons in the hypothalamus that contain the proopiomelanocortin peptide (POMC) have a vital role in the function of these neurons controlling feeding behavior in the mouse [17,48]. Loss of cilia from these neurons causes hyperphagia and obesity. To demonstrate the feasibility of using the Cilia^GFP^ mouse to aid in the study of cilia function in these neurons and to demonstrate expression restricted to a specific group of cells, we crossed the Cilia^GPF^ mouse with the POMC Cre line. The animals from this cross should express Sstr3::GFP specifically in the POMC neurons within the arcuate nucleus (ARC) of the hypothalamus (Figure 6A). In these POMC Cre; Cilia^GFP^mice, cilia labeling was detected within the ARC but not in other regions of the brain like the hippocampus (Figure 6A-D, N = 2). Staining sections of the hypothalamus with ACIII confirmed that Sstr3::GFP labeling was specific to primary cilia (Figure 6D).We did not quantify the efficiency of Cre recombination in the POMC neurons but, qualitatively, the distribution of neurons that had undergone recombination based on Sstr3::GFP expression appeared similar to or greater than that seen using the mT/mG Cre reporter mouse (Gt(ROSA)26Sor^tm4(ACTB-tdTomato,-EGFP)Luo^).

In addition to cell-type specific control of Sstr3::GFP expression, we could also induce expression at a specific time point in the life of a mouse. To demonstrate temporal control of the Cilia^GFP^ allele, we used the tamoxifen responsive Pdx1 Cre ER line. In adult mice, Pdx1 Cre ER is expressed in the β-cells of the pancreas. We harvested pancreata from mice three days after a series of tamoxifen injections (see Methods) and processed them for immunofluorescence. As shown in Figure 6E, many of the cilia in the islets were labeled with Sstr3::GFP. Primary cilia are present on islet cells and in the ducts of the pancreas as reported previously [49,50], and in agreement with known Pdx1 Cre expression, only the islets were labeled in these mice (Figure 6E). Specificity was confirmed with acetylated α-tubulin staining (Figure 6E) and the absence of Sstr3::GFP in the ducts as well as in animals lacking the Cre transgene animals was confirmed (Additional file 1: Figure S1A).

### Generation of the constitutively expressed Cilia^GFP-ON^ allele

**Figure 7:**
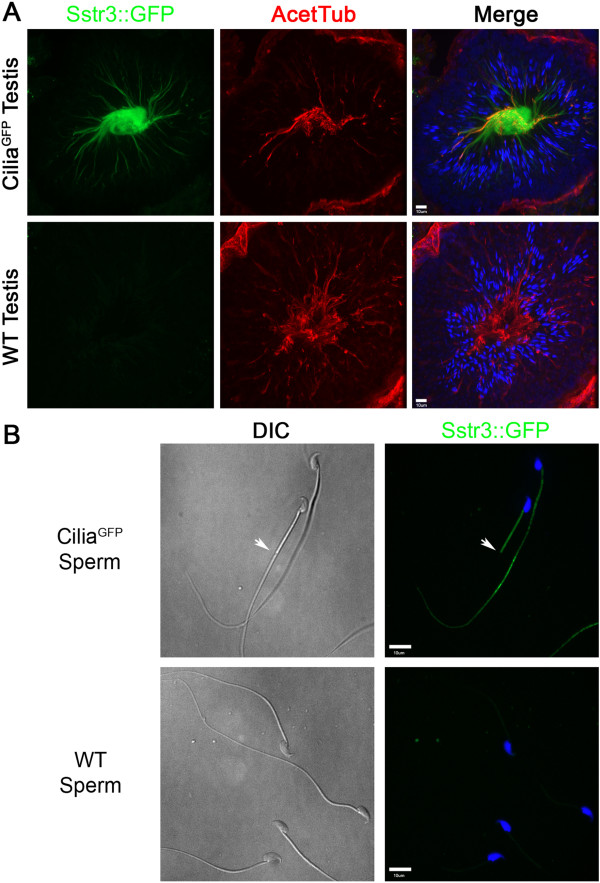
**Sstr3::GFP localization in testes and sperm.** (**A**) Sstr3::GFP localization in the mouse testes. Sstr3::GFP signal (green) can be seen in the sperm flagella in the center of the seminiferous tubules of Cilia^GFP-ON^ mice (upper panels), which are also positive for acetylated α-tubulin (red). Sstr3::GFP signal is absent in wild-type mouse testes (lower panels). (**B**) Sstr3::GFP signal is localized to the flagellum of Cilia^GFP^ mouse sperm but no signal is detected in wild-type mouse sperm. The arrow points to the example of a small subset of sperm where Sstr3::GFP appeared to be restricted to only the mid-piece of the flagellum. Nuclei are counterstained with Hoescht (blue).

To generate a line with constitutive expression of Sstr3::GFP, we utilized EIIa Cre that has a high frequency of germline Cre activity in females to remove the floxed stop sequence. In the offspring, Cre negative Cilia^GFP-ON^ mice were identified to establish the line. As observed in the inducible Cilia^GFP^ line, cilia were readily detected with the germline Cilia^GFP-ON^ mice. In heterozygous Cilia^GFP-ON^ females, we did not observe any overt deleterious effects of ectopic expression of Sstr3::GFP with the caveat that no in-depth behavioral analyses were performed; however, male Cilia^GFP-ON^ mice are sterile, even when carrying one copy of the Cilia^GFP-ON^ allele. The morphology of the testes in Cilia^GFP^ mice looks normal (Figure 7A) and the male mice do mate, as confirmed by vaginal plugs, and they produce sperm. However, isolation of sperm from the epididymis revealed they are immotile (Figure 7B and Additional files 6 and 7). The mature sperm flagella express Sstr3::GFP (Figure 7A, B) which seems to be interfering with their motility. We observed that a small subset of sperm had Sstr3::GFP only localizing to the mid-piece (Figure 7B), which may correspond to the small fraction of motile spermatozoa observed in the Cilia^GFP^samples (Additional file 7). We did not specifically isolate motile spermatozoa from Cilia^GFP^ mice to confirm SStr3::GFP localization; however, these sperm frequently displayed a hairpin bend right at the end of the mid-piece. This may indicate that the tail, without Sstr3::GFP, is motile while the Sstr3::GFP containing the mid-piece is not. It appears that spermatia with Sstr3::GFP localized completely throughout their flagella have no motility, which would indicate that Sstr3::GFP itself may be interfering with the molecular machinery necessary for sperm motility.

## Conclusions

Here we have developed a new tool for *in vivo* and *ex vivo* detection and visualization of mammalian cilia, the Cilia^GFP^ mouse. We have demonstrated that the Cilia^GFP^ mouse is functional, cilia specific, and that spatial and temporal control of expression is possible. In animals without Cre or without tamoxifen, no ciliary label was detected (Additional file 1: Figure S1A-C). In addition, there were a few tissues where ciliary labeling was not easily observable, such as the trachea and the motile cilia in the oviduct (Additional file 1: Figure S1D, E) of Cilia^GFP-ON^ mice. The cilia in these regions contained GFP but at a similar level as the rest of the cell membrane. The reason that cilia in these regions of the Cilia^GFP^ mouse were not highly labeled is unknown but it could be a result of the oviduct and tracheal epithelial cells having many more cilia per cell (approximately 150, Additional file 1: Figure S1D and E) [51] than ependymal cells (approximately 15 per cell, Figure 2B) [52] or choroid plexus cells (approximately 5 per cell, Figure 2C). Which might result in the dilution of the GFP signal among an increased number of cilia as has been shown for Smoothened in multi-ciliated cells [25].

Another caveat to using the Cilia^GFP^ mouse is that when the Cilia^GFP-ON^ allele is expressed in the testes, sperm from Cilia^GFP-ON^ males have impaired motility likely due to the localization of Sstr3::GFP in the flagella. This does raise the possibility that Sstr3 may be altering the function or motility of cilia in other tissues as well. This is an important factor to consider since a recent study has shown that over-expressing GPCRs like Sstr3 in cilia could affect the normal distribution of ciliary proteins and cilia morphology [28]. Aside from these exceptions, this mouse will facilitate the study of cilia in tissues where staining and imaging have been difficult, such as the brain and eye, and for studies that require live or *in situ* analysis.

There are multiple uses for this mouse model: for example, mammalian mutagenesis and *in vivo* pharmacological screens for factors that affect ciliogenesis, cilia length control and ciliary protein trafficking; to assess recovery of ciliary proteins by FRAP analysis; to analyze regulation of cilia motility; or to screen for suppressor mutations using known cilia mutants and assessing the restoration of ciliogenesis. Further, the Cilia^GFP^mouse can be used for assessing cilia loss when used in conjunction with floxed alleles of genes required for ciliogenesis, since Cre expression would simultaneously delete the floxed gene required for ciliogenesis and remove the floxed-stop cassette to induce Sstr3::GFP expression. Lastly, we have shown the utility of this model with *in situ* documentation of ciliary motion in the tubules of the kidney. Indeed, this observation indicates the Cilia^GFP^ mouse will be useful for *in vivo* mechanosensory studies that may provide important insights into how cilia dysfunction contributes to cyst development.

## Abbreviations

ACIII: Adenylate cyclase III; AcetTub: Acetylated α-tubulin; ARC: Arcuate nucleus; BSA: Bovine serum albumin; CC: Connecting cilium; CNS: Central nervous system; DABCO: 1,4-diazabicyclo[2.2.2]octane; DAPI: 4',6-diamidino-2-phenylindole; EIIa: Adenovirus early transcription region II DNA binding protein promoter; ES cells: Embryonic stem cells; FITC: Fluorescein isothiocyanate; GFP: Green fluorescent protein; INL: Inner nuclear layer; PBS: Phosphate-buffered serum; Pdx1: Pancreatic and Duodenal Homeobox 1; PFA: paraformaldehyde; POMC: Proopiomelanocortin; RCF: Relative Centrifugal Force; ROSA26: Reverse oriented splice acceptor β-galactosidase/neomycin 26; RT: Room temperature; Sstr3: Somatostatin receptor 3

## Competing interests

The authors have no financial interest to declare.

## Authors' contributions

AKO, EBM, NFB and BKY designed and performed experiments. AKO, EBM, NFB and BKY wrote the manuscript. MJC, CJH and PDB performed experiments. AKO and PH created the targeting construct. RAK created the mouse model. All authors read and approved the final manuscript.

## Supplementary Material

Additional file 1 **(A-C) Control sections showing no detectable Sstr3::GFP signal in Cre- animals.** No Sstr3::GFP signal (green) is seen in (A) pancreatic islets (B) the retina (C) and kidney tubules in Cilia^GFP^; Cre negative animals. The presence of cilia was confirmed with acetylated α-tubulin (red or purple). (D) Are presentative image of the trachea in a Cilia^GFP^mouse showing thatSstr3::GFP labeling in the motile cilia tufts is faint (acetylated tubulin, purple). The lamina propria (LP) shows strong autofluorescence, also seen in Sstr3::GFP negative mice. The chondrocytes (Ch) of the trachea show ciliary labeling (arrow). (E) Image of the oviduct showing faintSstr3::GFP labeling in the motile cilia tufts (acetylated tubulin, red). (F) Image of the retina showing colocalization of Sstr3::GFP expression and rhodopsin labeling in the rod calls. Arrows point to connecting cilia where GFP expression is strongest (outer nuclear layer, ONL). Click here for file

Additional file 2 ***Ex vivo*live imaging of ependymal cilia(same animal represented in Figure **2**B).** Synchronized wave-like movements of the cilia are easily observed. Images were captured and shown at 11 frames per second. Click here for file

Additional file 3 ***In situ *video microscopy of a kidney tubule captured at a high frame rate displaying no ciliary oscillation.** With high volume of glomerular filtration cilia in the tubules lay parallel to the surface of the epithelium with a common deflection point located near the base of the cilium. Occasionally, a cilium within the tubule will reverse its position (shown at 26 fps). Click here for file

Additional file 4 ***In situ *video microscopy of a kidney tubule captured at a high frame rate displaying ciliary oscillation.** Cilia in the tubule oscillate synchronously back and forth within the tubules in the field (shown at 26 fps). Click here for file

Additional file 5 ***In situ *imaging of ciliary movement within a tubule before and after death. Synchronized movement of the cilium terminates with death of the animal.** Further these cilia extend into the tubule lumen supporting a passive rather than motor driven mechanism of movement (shown at 11 fps). Click here for file

Additional file 6 **Live sperm collected from a wild-type mouse.** Differential interference contrast time-lapse movie (10 fps) showing movement of wild-type mouse sperm. Click here for file

Additional file 7 **Live sperm collected from a Cilia**^**GFP **^**mouse.** Differential interference contrast time-lapse movie (10 fps) showing lack of movement or abnormal movement of sperm from Cilia^GFP^mouse. Click here for file
