# Supplementary figures and images for: An inducible CiliaGFP mouse model for in vivo visualization and analysis of cilia in live tissue
Source: Cilia. 2013 Jul 3;2:8. doi: 10.1186/2046-2530-2-8 (PMC3700774; doi:10.1186/2046-2530-2-8)

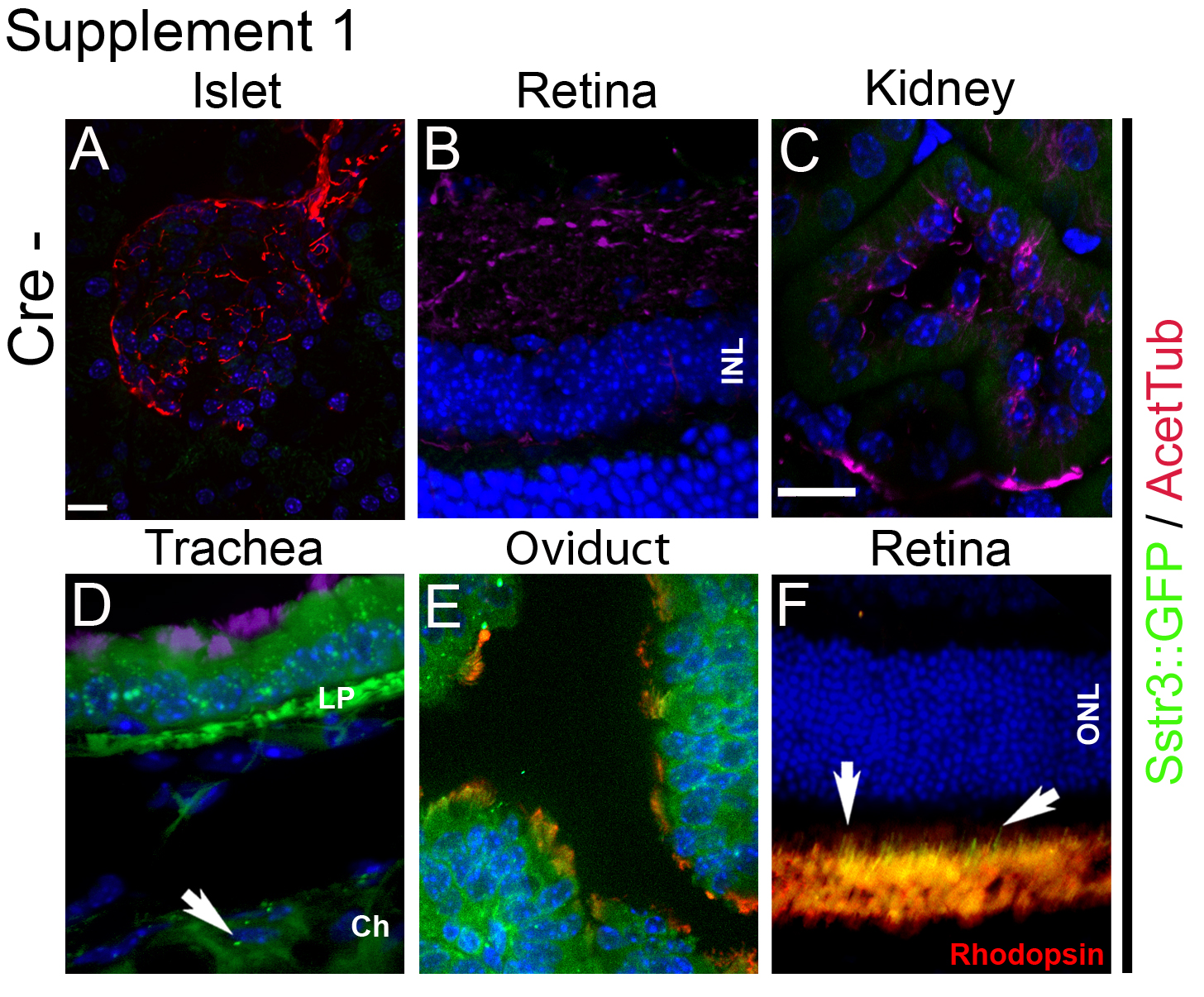

Supplement: Additional file 1 — (A-C) Control sections showing no detectable Sstr3::GFP signal in Cre- animals. No Sstr3::GFP signal (green) is seen in (A) pancreatic islets (B) the retina (C) and kidney tubules in CiliaGFP; Cre negative animals. The presence of cilia was confirmed with acetylated α-tubulin (red or purple). (D) Are presentative image of the trachea in a CiliaGFPmouse showing thatSstr3::GFP labeling in the motile cilia tufts is faint (acetylated tubulin, purple). The lamina propria (LP) shows strong autofluorescence, also seen in Sstr3::GFP negative mice. The chondrocytes (Ch) of the trachea show ciliary labeling (arrow). (E) Image of the oviduct showing faintSstr3::GFP labeling in the motile cilia tufts (acetylated tubulin, red). (F) Image of the retina showing colocalization of Sstr3::GFP expression and rhodopsin labeling in the rod calls. Arrows point to connecting cilia where GFP expression is strongest (outer nuclear layer, ONL). [file 2046-2530-2-8-S1.jpeg]
